# Roles of osteoclasts in alveolar bone remodeling

**DOI:** 10.1002/dvg.23490

**Published:** 2022-06-27

**Authors:** Maiko Omi, Yuji Mishina

**Affiliations:** ^1^ Department of Biologic and Materials Sciences & Prosthodontics University of Michigan School of Dentistry Ann Arbor Michigan USA

**Keywords:** alveolar bone, bone remodeling, bone resorption, jawbone, mechanical stress, osteoclast, periodontitis

## Abstract

Osteoclasts are large multinucleated cells from hematopoietic origin and are responsible for bone resorption. A balance between osteoclastic bone resorption and osteoblastic bone formation is critical to maintain bone homeostasis. The alveolar bone, also called the alveolar process, is the part of the jawbone that holds the teeth and supports oral functions. It differs from other skeletal bones in several aspects: its embryonic cellular origin, the form of ossification, and the presence of teeth and periodontal tissues; hence, understanding the unique characteristic of the alveolar bone remodeling is important to maintain oral homeostasis. Excessive osteoclastic bone resorption is one of the prominent features of bone diseases in the jaw such as periodontitis. Therefore, inhibiting osteoclast formation and bone resorptive process has been the target of therapeutic intervention. Understanding the mechanisms of osteoclastic bone resorption is critical for the effective treatment of bone diseases in the jaw. In this review, we discuss basic principles of alveolar bone remodeling with a specific focus on the osteoclastic bone resorptive process and its unique functions in the alveolar bone. Lastly, we provide perspectives on osteoclast‐targeted therapies and regenerative approaches associated with bone diseases in the jaw.

## 1 INTRODUCTION

Our skeletal system includes more than 200 bones which provide a rigid framework to support the body and serve as a reservoir of calcium. The jawbone is morphologically and functionally different from other skeletal bones. It is originated from migrating cranial neural crest cells and forms through primarily intramembranous ossification which differs from the axial and appendicular skeletons that arise from the mesoderm and undergo endochondral ossification. The alveolar bone is the part of the jawbone that displays anatomically and functionally unique characteristics. It retains the teeth through periodontal fibers called periodontal ligament (PDL) and is constantly subjected to strains with occlusal forces during mastication. Since this complex structure develops during tooth eruption, it does not regenerate easily once the tooth is lost. Therefore, it is crucial to understand the unique tissue structure and biological function of the alveolar bone for treating diseases and regenerating lost tissues.

Bone remodeling is a process whereby bone is constantly removed and replaced with new bone. These two distinct events, resorption and formation, are well coordinated in a temporal and spatial manner to maintain skeletal integrity; their dysregulation is associated with bone diseases in the jaw. Osteoclasts are multinucleated cells derived from the hematopoietic lineage and have the ability to resorb bone matrix. Osteoclastic bone resorption is controlled by several mediators such as RANKL and OPG. Inhibiting the mediators of osteoclast differentiation and activity to resorb bone can result in an increase in bone mass. For this reason, it is important to understand the mechanisms of osteoclastic bone resorption specifically associated with bone diseases in the jaw. This review will address the mechanism of alveolar bone remodeling with a specific focus on recent advances in understanding the mechanisms of osteoclast differentiation and function. Lastly, we provide perspectives on osteoclast‐targeted therapies and regenerative approaches associated with bone diseases in the jaw.

## 2 ALVEOLAR BONE DEVELOPMENT AND HOMEOSTASIS

Bones are derived from three distinct embryonic origins: the paraxial mesoderm, lateral plate mesoderm, and neural crest. The somites developed from the paraxial mesoderm generate the axial skeleton and the cephalic mesoderm, a part of the paraxial mesoderm, generates the posterior cranial bones, the lateral plate mesoderm generates the limb skeleton, and the cranial neural crest generates the anterior craniofacial bones (Bronner‐Fraser, 1994; Cohn & Tickle, 1996; Mishina & Snider, 2014; Tam & Trainor, 1994). The bone is formed through two specific ossification processes: intramembranous ossification and endochondral ossification (Erlebacher, Filvaroff, Gitelman, & Derynck, 1995; Kronenberg, 2003). The intramembranous ossification is the process by which mesenchymal cells directly differentiate into osteoblasts and produce bone matrix. The flat bones including the bones of the skull, maxilla, and mandible develop through this mechanism. Most bones such as long bones and axial bones, on the other hand, develop through endochondral ossification by which mesenchymal cells condense and form cartilage primordia. This cartilaginous template is subsequently replaced by bones.

**FIGURE 1:**
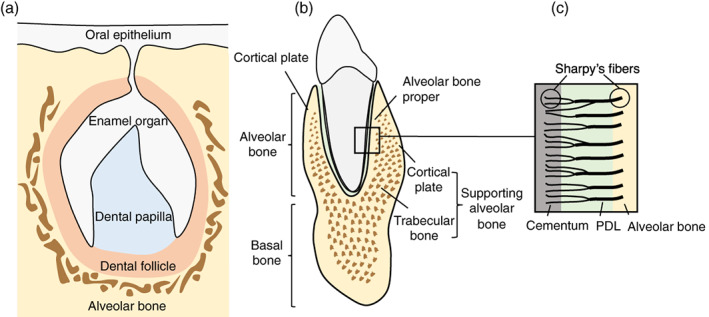
Schematic representation of alveolar bone development. The enamel organ, dental papilla, and dental follicle constitute the tooth germ and give rise to the essential structures of the tooth and supporting tissues: the enamel, dentin–pulp complex, and periodontium including cementum, periodontal ligament (PDL), and alveolar bone, respectively (a). The alveolar bone (alveolar process) rests on the basal bone of the mandible and maxilla, and consists of alveolar bone proper and supporting bone. The trabecular bone is located between alveolar bone proper and plates of cortical bone (b). Sharpey's fibers are bundles of PDL collagen fibers embedded in the alveolar bone proper and cementum at a right angle (c)

The alveolar bone (also known as the alveolar process) in the jaw forms through intramembranous ossification and originates from the cranial neural crest‐derived dental mesenchyme consisting of the dental follicle which surrounds the developing tooth germ (Figure 1a). During tooth development, the dental papilla gives rise to odontoblasts and the dental pulp, while the dental follicle gives rise to cementum, periodontal ligament (PDL), and alveolar bone (Ten Cate & Mills, 1972; Ten Cate, Mills, & Solomon, 1971). At the late bell stage, bony septa and bony bridges start to form, and separate each tooth germ from another. The tooth germs within bony compartments continuously move in various directions to adjust to the growing jaws, which induces bone remodeling through bone resorption followed by bone formation. Like bone in other sites, alveolar bone functions as a mineralized supporting tissue, providing mechanical strength, an environment for bone marrow, and serving as a reservoir for calcium ions.

The alveolar bone is a complex structure that rests on the basal bone of the maxilla and mandible and forms the tooth sockets (Hassell, 1993). It is attached to the tooth through connective tissue fibers called PDL. The general functions of the alveolar bone are to hold the roots of teeth, and to absorb and distribute occlusal forces generated by oral functions (Cho & Garant, 2000). There are two major parts of the alveolar bone: alveolar bone proper and supporting alveolar bone (Chiego, 2018) (Figure 1b). The bone of the tooth socket is a dense cortical plate named alveolar bone proper (also known as lamina dura or cribriform plate) and provides attachment for the PDL. It is also called as bundle bone because Sharpey's fibers, a portion of the fibers of the PDL, are inserted at a right angle to the tooth and bone surface (Chiego, 2018) (Figure 1c). This bundle bone is gradually resorbed on the marrow space side and replaced by lamellar bone following tooth extraction (Cardaropoli, Araujo, & Lindhe, 2003; Lindhe et al., 2013). The supporting alveolar bone consists of cortical plates and trabecular bone. The cortical plates locate on the labial and lingual surfaces of the alveolar bone. The trabecular bone (also called cancellous or spongy bone) is located between the alveolar proper and the cortical plates. Since the alveolar bone gradually develops during the formation and eruption of the teeth and diminishes in height with tooth loss, they are tooth‐dependent bony structures (Cho & Garant, 2000). Therefore, the size, shape, location, and function of the teeth influence the overall structure of the alveolar bone.

## 3 OSTEOCLASTIC BONE RESORPTION

**FIGURE 2:**
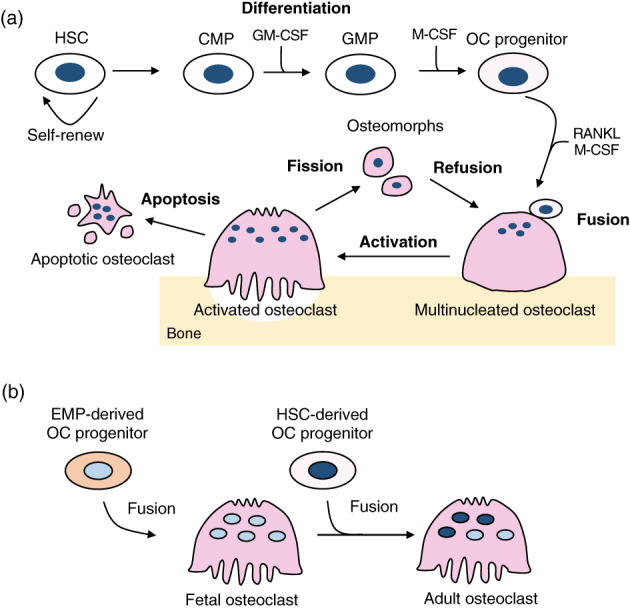
Schematic representation of osteoclast origin and differentiation. The hematopoietic stem cells (HSCs) give rise to common myeloid progenitors (CMPs) and differentiate into granulocyte/macrophage progenitors (GMPs) by stimulation with granulocyte/macrophage stimulating factor (GM‐CSF). GMPs further differentiate into monocytes/macrophage lineage by M‐CSF stimulation and become osteoclast (OC) progenitors. OC progenitors enter the blood circulation and migrate toward bone surfaces where they fuse and become multinucleated osteoclasts upon stimulation with M‐CSF and receptor activator of NF‐κB ligand (RANKL). Activated mature osteoclasts resorb bone matrix and undergo either apoptosis or recycling via osteomorphs (a). During embryonic development, yolk sac erythroid–myeloid progenitors (EMPs) differentiate into OC progenitors and become multinucleated osteoclasts. In postnatal life, these precursors are gradually replaced by HSC‐derived OC progenitors that fuse with EMP‐derived osteoclasts (b)

Osteoclasts are large multinucleated cells that originated from hematopoietic stem cells (HSCs) and are responsible for bone resorption (Figure 2a). The HSCs give rise to common myeloid progenitors (CMPs) which differentiate into granulocyte/macrophage progenitors (GMPs) by stimulation with granulocyte/macrophage stimulating factor (GM‐CSF) (Seita & Weissman, 2010). GMPs further differentiate into monocytes/macrophage lineage and become osteoclast progenitors, which enter the blood circulation and migrate toward bone surfaces where they fuse and become multinucleated osteoclasts (Seita & Weissman, 2010). Recent studies provided new evidence that erythro‐myeloid progenitors (EMPs) in the yolk sac serve as precursors for osteoclasts during embryonic development (Jacome‐Galarza et al., 2019; Yahara et al., 2020). The early EMPs appear around E7‐E7.5 in the mouse yolk sac and differentiate into yolk‐sac macrophages which give rise to tissue‐resident macrophage populations such as microglia in the brain (Ginhoux et al., 2010). The late EMPs appear around E8.25‐E9 in the yolk sac and migrate to the fetal liver to produce fetal liver monocytes (Gomez Perdiguero et al., 2015; Mass et al., 2016). These EMP‐derived monocytes/macrophages give rise to embryonic osteoclasts, forming the bone marrow cavity around E15.5 to establish hematopoiesis during development (Yahara, Nguyen, Ishikawa, Kamei, & Alman, 2022). These EMP‐derived osteoclasts are gradually replaced by HSC‐derived osteoclast progenitors during postnatal bone remodeling (Jacome‐Galarza et al., 2019), likely through replenishment through continuous fusion with HSC‐derived progenitors (Figure 2b). Indeed, the EMP‐derived osteoclast precursors can travel through the bloodstream to the injury site and differentiate into mature osteoclasts during fracture healing (Yahara et al., 2020). Although these EMP‐derived osteoclasts are observed 6 months after birth, it is uncertain whether these osteoclasts stay alive throughout the entire lifespan, and is an area of active investigation. It has been suggested that dendric cells which are also derived from CMPs can become precursors for osteoclasts in the pathological condition such as inflammatory bone loss (Speziani et al., 2007). Yet, it is unclear whether distinct origins of osteoclasts have the same resorption activity and how they are involved in bone remodeling at different skeletal sites.

Activation and differentiation of osteoclasts are controlled by two essential cytokines: macrophage colony‐stimulating factor (M‐CSF) and receptor activator of nuclear factor‐κB ligand (RANKL; Figure 2a). Binding of M‐CSF (also known as CSF‐1) to its receptor, c‐Fms, on myeloid progenitors supports the survival and proliferation of these progenitors (Felix et al., 1990; Udagawa et al., 1990). M‐CSF also induces the expression of receptor activator of NF‐κB (RANK) and thus facilitates RANKL/RANK signaling in osteoclasts (Arai et al., 1999). The binding of RANKL to RANK activates NF‐kB and MAPK signaling pathways, resulting in the induction of two key osteoclastic transcription factors, c‐Fos and nuclear factor‐activated T cells c1 (NFATc1) (Aliprantis et al., 2008; Grigoriadis et al., 1994; Xing et al., 2002). Activation of these factors, in turn, regulates osteoclast‐specific genes such as tartrate‐resistant acid phosphatase (TRAP) and cathepsin K, which are required for osteoclast differentiation. Therefore, RANKL/RANK system is indispensable for osteoclastogenesis and bone remodeling as deletion of *Rankl* in mice leads to severe osteopetrosis (Kong et al., 1999). Interestingly, these mice also exhibit impaired tooth eruption, indicating that bone resorption by osteoclasts is required for successful tooth eruption. Inflammatory cytokines such as tumor necrosis factor‐α (TNF‐α) and interleukin‐1 (IL‐1) potentiate RANKL‐induced osteoclastogenesis (Luo, Li, Li, Wang, & Zhang, 2018; Quinn, Horwood, Elliott, Gillespie, & Martin, 2000). Although these inflammatory cytokines do not substitute for RANKL in physiological conditions (Li et al., 2000), they prompt robust osteoclastogenesis in inflammatory conditions even without elevation of RANKL levels (Kobayashi et al., 2000; Lam et al., 2000).

M‐CSF and RANKL are produced by several different types of cells such as bone marrow stromal cells, osteoblasts, osteocytes, and chondrocytes, which are crucial for physiological bone remodeling (Udagawa et al., 1990; Usui et al., 2008; Zhao, Zhang, Harris, Ahuja, & Bonewald, 2002). In alveolar bone remodeling, gingival fibroblasts and PDL cells produce RANKL/OPG and thus control the activity of osteoclasts (Kim, Handa, Iida, & Yoshida, 2007; Shiotani, Shibasaki, & Sasaki, 2001). Immune cells such as B cells and T cells also produce M‐CSF and RANKL to regulate osteoclastogenesis. Interestingly, RANKL knockout mice also carrying transgenic RANKL gene expressed in B cells and T cells restore osteoclast formation in long bones; however, the “rescued” mice still do not display signs of tooth eruption (Odgren et al., 2003). In pathological conditions such as periodontitis, however, RANKL produced from B cells in gingival tissues promotes alveolar bone resorption (Settem, Honma, Chinthamani, Kawai, & Sharma, 2021). These results suggested that RANKL from different types of cells plays a role in osteoclast functions depending on anatomical locations of bones and under different developmental or pathophysiologic conditions.

Cell fusion is an important process in the formation of osteoclasts and acquiring a bone resorption activity. The fusion of osteoclast progenitors only occurs on the bone surface but not in the bone marrow (Baron, Neff, Tran Van, Nefussi, & Vignery, 1986). It has been found that existing osteoclasts on the bone surface become multinucleated by continuous fusion with a circulating mononucleated osteoclast progenitor (Jacome‐Galarza et al., 2019; Levaot et al., 2015). Interestingly, cell fusion events usually occur between a mobile and an immobile fusion partner (Soe et al., 2019; Soe, Hobolt‐Pedersen, & Delaisse, 2015). These results suggested the importance of osteoclast mobility and nuclearity for finding the right fusion partner. Several proteins such as dendritic‐cell‐specific transmembrane protein (DC‐STAMP), osteoclast stimulatory transmembrane protein (OC‐STAMP), and Atp6v0d2 are involved in the fusion process. Interestingly, deletion of DC‐STAMP in mice exhibits osteopetrosis due to a defect in osteoclast fusion while cells from these mice can still resorb bone matrix with low resorption activity (Yagi et al., 2005), suggesting that cell fusion is not an essential step for bone resorption but affects the efficiency of osteoclast activity.

**FIGURE 3:**
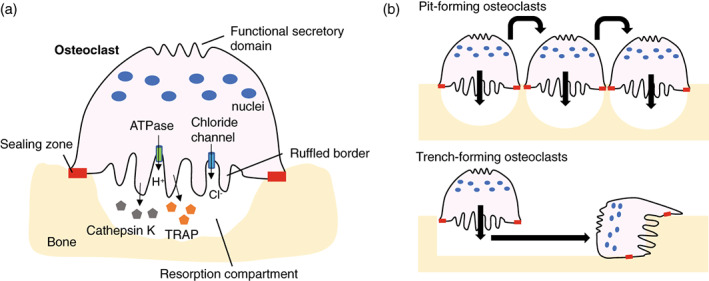
Schematic representation of bone resorption by osteoclasts. Activated osteoclasts form the sealing zone that surrounds the ruffled border and defines the area of bone surface to be resorbed. During bone resorption, H^+^ (protons) and chloride ions are transported through the Vacuolar H^+^‐ATPase and chloride channel localized on the ruffled border, respectively. Degradation of bone matrix is mediated by enzymes such as cathepsin K and tartrate‐resistant acid phosphatase (TRAP). Matrix degradation products are endocytosed from the ruffled border and released from the functional secretory domain (a). Osteoclasts resorb bone in two different modes: the intermittent and stationary resorption mode resulting in rounded resorption cavities termed pits, and the continuous resorption mode where the osteoclast moves during resorption resulting in deep elongated cavities termed trenches (b)

Osteoclastic bone resorption is achieved by the dissolution of hydroxyapatite by secretion of acid, followed by degradation of the organic component of bone matrix by proteolysis (Rucci & Teti, 2016). The formation of unique cytoskeletal structures termed the “sealing zone” and the “ruffled border” are required for efficient bone resorption (Takito, Inoue, & Nakamura, 2018). The resorption compartment is formed by the attachment of osteoclasts to the bone surface through the sealing zone (Figure 3a). The ruffled border localized within the sealing zone transports protons and proteases into the resorption compartment to demineralize and degrade the collagen I‐rich matrix, respectively. Vacuolar H^+^‐adenosine triphosphatase (H^+^‐ATPase) located in the ruffled border membrane transport protons to acidify the resorption compartment (Blair, Teitelbaum, Ghiselli, & Gluck, 1989; Li, Chen, Liang, Li, & Stashenko, 1999). Chloride ions are also transported into the resorption compartment via a chloride channel present in the ruffled border membrane to maintain electroneutrality (Kornak et al., 2001; Lange, Wartosch, Jentsch, & Fuhrmann, 2006; Schlesinger, Blair, Teitelbaum, & Edwards, 1997). The degradation of collagen I‐rich matrix is mediated by a lysosomal protease, cathepsin K (Drake et al., 1996; Gowen et al., 1999). The degraded products such as collagen and other protein fragments, calcium and phosphate within the resorption compartment, are endocytosed and released from the functional secretory domain (FSD) at the basolateral membrane of the osteoclast (Nesbitt & Horton, 1997; Salo, Lehenkari, Mulari, Metsikko, & Vaananen, 1997). It was found that osteoclasts resorb bone and form either circular pits or long trenches into bone matrix (Soe & Delaisse, 2017; Figure 3b). The trench‐forming osteoclasts are more aggressive and continuous compared to pit‐forming osteoclasts (Borggaard, Pirapaharan, Delaisse, & Soe, 2020; Merrild et al., 2015). The prevalence of these distinct resorption modes may be due to different precursor origins, different skeletal sites, gender, or levels of systemic and local regulators. Generally, trabecular bone has a large surface exposed to the bone marrow and blood flow, and the turnover rate is higher than in cortical bone (Parfitt, 2002). Indeed, bone resorption occurs along the bone surface in the trabecular bone, whereas bone resorption takes place through tunnels in the cortical bone (Eriksen, 2010), suggesting that osteoclasts in the trabecular bone may be a trench‐forming type while those in the cortical bone are a pit‐forming type. Yet, it is unclear whether osteoclasts in the alveolar bone exhibit two distinct modes of bone resorption and what molecular mechanisms are responsible for the specification of trench‐ and pit‐forming osteoclasts.

It has been considered that osteoclasts undergo apoptosis in a relatively short time after they accomplish bone resorption as their life span in a healthy human adult is approximately 2 weeks in the marrow (Manolagas, 2000). Recent technological advances such as intravital imaging in bone have provided new insights into osteoclast biology. It was found that osteoclasts have an alternative cell fate in which they undergo fission into daughter cells called osteomorphs (McDonald et al., 2021). Interestingly, these fission products are found in circulation and bone marrow and re‐fuse with other osteoclasts on the bone surface, a process referred to as osteoclast recycling. Further studies are needed to address whether osteoclast recycling occurs during alveolar bone remodeling and underlies jawbone diseases such as periodontitis and osteonecrosis of the jaw (ONJ), which may reveal further insights into the cellular dynamics of alveolar bone remodeling.

## 4 ROLES OF OSTEOCLASTS IN ALVEOLAR BONE REMODELING

**FIGURE 4:**
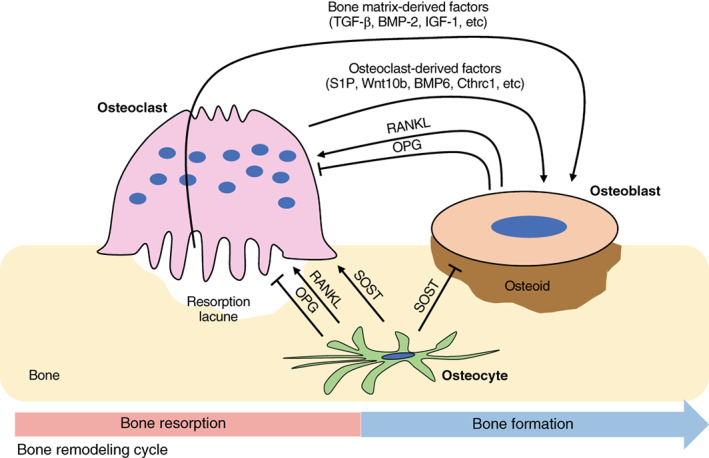
Schematic representation of cellular interactions between osteoclasts, osteoblasts, and osteocytes in bone remodeling. Osteoblasts and osteocytes express RANKL and osteoprotegerin (OPG). RANKL binding to RANK receptor on the osteoclasts leads to differentiation and activation of osteoclasts. OPG acts as a decoy receptor for RANKL and thus inhibits osteoclast differentiation. Osteocytes inhibit bone formation and promote bone resorption via Sclerostin (SOST). Osteoclasts regulate migration and activity of osteoblasts through factors secreted from osteoclasts themselves and bone resorption cavities

Bone remodeling is an essential process to maintain mineral homeostasis and to repair damaged bone. In adults, it has been estimated that 10% of bone is replaced every year, which is crucial for maintenance of the skeleton system (Manolagas, 2000). The remodeling process involves coordinated action of bone cells including osteoclasts, osteoblasts, and osteocytes (Figure 4). The amount of bone resorbed by osteoclasts matches the amount of newly formed bone by osteoblasts and thus the net bone mass is maintained. This system is known as *coupling* and osteoclasts play a critical role in controlling the balance of bone formation and resorption (Martin & Sims, 2005). For example, osteoclast‐derived secreted products and bone resorption‐derived coupling activities enhance bone formation through either the recruitment of pre‐osteoblasts or the direct stimulation of osteoblastic bone formation. Yet, it is unknown whether such coupling factors exhibit similar functions in the different bony sites and whether particular factors are responsible for osteoclast–osteoblast coupling in the alveolar bone.

It has been considered that all osteoclasts have similar functional properties; however, several studies suggest differences in the activity of the osteoclasts from different skeletal sites. For instance, bone marrow cells from the jawbone generate more osteoclasts and display higher resorption activity than those from the long bone in culture (Chaichanasakul, Kang, Bezouglaia, Aghaloo, & Tetradis, 2014; de Souza Faloni et al., 2011). Interestingly, long bone cells generate more osteoclasts than jawbone cells when cultured on the bone slice, while the jawbone cells generate more osteoclasts than the long bone cells when cultured on the dentin slice (Azari, Schoenmaker, de Souza Faloni, Everts, & de Vries, 2011), suggesting that differences in osteoclasts and their activity are likely related to the differences in the substrate they resorb. Since composition of ECM differs between the jawbone and the long bone (Matsuura et al., 2014), such differences in ECM may provide an explanation for the unique characteristics of osteoclasts in the jawbone. It is also possible that these differences in osteoclasts from different sites of the body are due to cell‐intrinsic differences. It has been shown that resorption by calvarial osteoclasts depends on the activity of two major classes of proteolytic enzymes, the cysteine proteinases and matrix metalloproteinases (MMPs), whereas resorption by long bone osteoclasts depends primarily on the activity of the cysteine proteinases (Everts et al., 1999). Since inhibition of MMPs does not affect resorption activity of long bone osteoclasts cultured on bone slices either from long bones or skull while resorption by calvarial osteoclasts is inhibited, it is suggested that these differences may be intrinsically related to the osteoclast. It is not unknown yet if osteoclasts from the alveolar bone exhibit differences in the level of MMP activity compared to those from other bones and if the cell‐intrinsic differences affect their activities.

**FIGURE 5:**
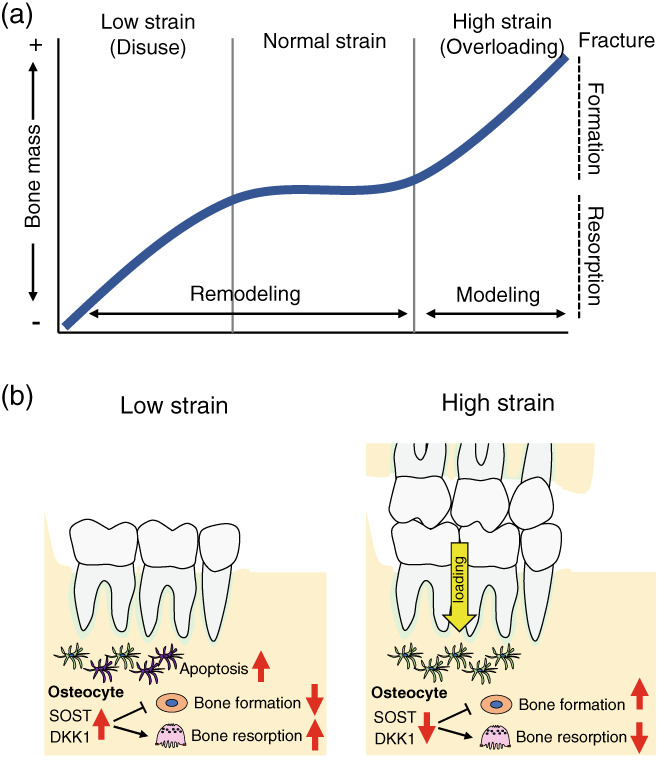
Schematic representation of alveolar bone remodeling during mastication. Mechanostat model of functional adaptation of bone. Above the certain threshold levels for strain, bone formation modeling which is independent of bone resorption occurs to increase bone mass. A lower than the certain threshold level for strain leads to increased bone remodeling and resorption, resulting in decreased bone mass. In the normal loading range, bone formation and bone resorption are balanced, and total bone mass is unchanged (a). Sclerostin (SOST) and DKK1 inhibit osteoblast differentiation and stimulate osteoclastogenesis. Lower mechanical loading results in osteocyte apoptosis and increases in sclerostin production, leading to increased bone resorption. Higher mechanical loading by occlusal forces during mastication reduces sclerostin production from osteocytes, which decreases bone resorption and accelerates new bone formation in the alveolar bone (b)

It has been demonstrated that turnover in the alveolar bone is faster than that in the other skeletal bones. For instance, the bone formation rate (BFR) in the alveolar bone of the maxilla (19.1%/year) and mandibular (36.9%/year) is 3‐ to 6‐fold higher than the femur (6.4%/year) in dogs (Huja, Fernandez, Hill, & Li, 2006). The reason for this elevated bone turnover in alveolar bone is not clearly understood. It is possible that one of the reasons for the fast bone remodeling is mechanical stimulation during mastication (Omi & Mishina, 2020). Bone mass and architecture change as a result of adaptation to mechanical forces. For example, bone mass is decreased below a certain threshold strain level, as is observed in situations of disuse while bone mass increases when strains are greater than a certain level to increase load‐bearing strength (Frost, 1987). This mechanostat model describes critical roles of bone modeling and remodeling for functional adaptation of bone (Figure 5a). This model is applied to the long bones in response to static loading; hence, it is uncertain if it is applicable to the alveolar bone in response to dynamic loading as seen in the mastication. It has been shown that bone is more sensitive to dynamic strain changes rather than static strain (Lanyon & Rubin, 1984; Robling, Duijvelaar, Geevers, Ohashi, & Turner, 2001). For instance, dynamic, but not static loading, increases bone formation, resulting in increased bone mass (Lanyon & Rubin, 1984; Li et al., 2013). Indeed, dynamic loading decreases RANKL expression from osteoprogenitor stromal cells and suppresses osteoclastogenesis (Rubin, Murphy, Fan, Goldschmidt, & Taylor, 2002; Rubin, Murphy, Nanes, & Fan, 2000). The mechanostat thresholds therefore relate to strain magnitude, duration, and frequency, and each bone may have different thresholds to induce bone modeling and remodeling.

There are several animal studies investigating the effect of mechanical loading induced by mastication on alveolar bone mass. For instance, a reduction of occlusal forces by soft diet leads to decreased bone mineral density and bone volume in the alveolar bone (Mavropoulos, Kiliaridis, Bresin, & Ammann, 2004). Additionally, loss of occlusal forces by inserting a bite‐raising appliance also decreases trabecular bone volume in the alveolar bone (Liu, Jin, & Li, 2015). Osteocytes embedded in the bone matrix are the primary mechanosensitive cells involved in the transduction of mechanical stress into a biological response (Klein‐Nulend et al., 1995; Tatsumi et al., 2007). Osteocytes exert their effects on bone remodeling via direct cell–cell contact and by releasing soluble factors that control the activity of osteoclasts and osteoblasts. Particularly, sclerostin and Dickkopf‐related protein 1 (DKK‐1) produced from osteocytes play critical roles in the regulation of bone remodeling (Winkler et al., 2003). These factors inhibit the Wnt/β‐catenin pathway and decrease bone formation (Winkler et al., 2003). Sclerostin also promotes osteoclastogenesis in a RANKL‐dependent manner (Wijenayaka et al., 2011). Mechanical loading decreases sclerostin and DKK‐1 production from osteocytes and increases bone formation (Robling et al., 2008; Tu et al., 2012). Similarly, increased mechanical loading by a hard diet suppresses sclerostin expression in the alveolar bone (Inoue et al., 2019), suggesting that mechanical loading during mastication inhibits sclerostin production from osteocytes, leading to a decrease in bone resorption and an increase in bone formation (Figure 5b). By contrast, mechanical unloading increases sclerostin production, leading to reduced activity of osteoblasts and viability of osteoblasts and osteocytes (Lin et al., 2009). Moreover, *Sost*‐deficient mice are resistant to mechanical unloading‐induced bone loss (Lin et al., 2009), suggesting that disuse‐mediated bone loss may also be dependent on sclerostin. Indeed, osteocyte apoptosis is necessary for bone resorption to be initiated when in a state of disuse (Aguirre et al., 2006) (Figure 5b). However, it was found that osteocyte ablation using diphtheria toxin (DT) receptor (DTR) transgenic mice results in an increase in osteoclast numbers in femur, but not in alveolar bone (Matsumoto, Iimura, Ogura, Moriyama, & Yamaguchi, 2013; Tatsumi et al., 2007), suggesting that osteocytes from different anatomical locations of bone may exhibit different actions on osteoclasts and osteoblasts.

Mechanical stress also plays an important role in the pathogenesis of periodontal disease. For example, strong forces induced by occlusal trauma increase RANKL‐producing cells in periodontium and accelerate alveolar bone resorption in periodontitis (Yoshinaga, Ukai, Abe, & Hara, 2007). The mechanical loading also enhances the production of cytokines in PDL cells induced by periodontal bacteria (Yamamoto et al., 2011). Furthermore, mechanical stimulation during mastication in periodontitis induces IL‐6 production from gingival epithelial cells and promotes the accumulation of Th17 cells in the gingival mucosa, resulting in inflammatory alveolar bone loss associated with increased bone resorption (Dutzan et al., 2017). Although mechanical loading is thought to induce anabolic effects for bone diseases such as osteoporosis, it is not the case in periodontitis. Understanding the molecular basis of cellular responses to mechanical stress in the alveolar bone both in physiological and pathological conditions will provide new insights into the unique features of alveolar bone remodeling.

## 5 OSTEOCLAST‐RELATED DISEASES, CURRENT THERAPEUTICS, AND REGENERATIVE APPROACHES

### 5.1 Osteoporosis

Osteoporosis is a serious public health problem characterized by reduced bone mass which is associated with excessive osteoclast activity. Medications to treat osteoporosis are categorized as either antiresorptive (i.e., bisphosphonates, denosumab) or anabolic (i.e., teriparatide) (Awasthi, Mani, Singh, & Gupta, 2018; Chen & Sambrook, 2011). Antiresorptive medications primarily inhibit the activity of osteoclasts while anabolic medications promote the activity of osteoblasts and increase bone formation more than bone resorption. The most widely used medications for the treatment of osteoporosis are antiresorptive medications such as bisphosphonates and denosumab. Bisphosphonates bind with high affinity to the hydroxyapatite crystals of the bone and inhibit osteoclast resorption of the bone, leading to a decrease in bone turnover and net gain in bone mass (Frith, Monkkonen, Blackburn, Russell, & Rogers, 1997; Roelofs et al., 2012). Denosumab is a human monoclonal antibody that inhibits RANKL and decreases osteoclastic bone resorption (Cummings et al., 2009). These medications are effective for preventing osteoclastic bone resorption and reducing osteoclast number; however, they do not promote osteoblastic bone formation because osteoclast‐derived coupling factors, which are required for promoting the activity of osteoblasts, are lost (Bilezikian et al., 2009; Jensen, Andersen, Chavassieux, Roux, & Delaisse, 2021). By contrast, odanacatib, a small‐molecule inhibitor of cathepsin K, reduces bone resorption with a minimal decrease in bone formation rate in osteoporotic patients (Duong et al., 2016; Rizzoli et al., 2016). However, the development of odanacatib was terminated due to an increased incidence of stroke (Mullard, 2016).

Both bisphosphonates and denosumab are reported to be associated with a rare jawbone disease called medication‐related ONJ, defined as exposed bone in the maxillofacial region for more than 8 weeks (Ruggiero et al., 2022). It is typically observed after the extraction of teeth with severe periodontal or periapical infections in patients receiving prolonged antiresorptive therapy (Dodson, 2015; Filleul, Crompot, & Saussez, 2010), suggesting that bacterial infection, inflammation, and/or trauma may trigger ONJ if the patients are under antiresorptive treatment during a prolonged period. Several animal studies demonstrate that osteoclasts in the alveolar bone display distinct responses to antiresorptive agents compared to other bones. For instance, osteoclast precursors from the alveolar bone internalize more bisphosphonates than those in the long bone in vitro (Vermeer et al., 2013). Additionally, the accumulation of bisphosphonates in the alveolar bone is higher than that in the ilium in the ONJ mouse model (Su, Feng, Han, & Zhao, 2015). Interestingly, osteoclasts in the alveolar bone display higher expression of the anti‐apoptotic genes such as *Bcl‐2* and *Bcl‐xL* than those in the long bone (Vermeer et al., 2013). These unique characteristics of the osteoclasts in the alveolar bone may explain why ONJ occurs frequently in the jaw. Although bisphosphonates can suppress osteoclastic bone resorption, the long‐term use of bisphosphonates, particularly nitrogen‐containing bisphosphonates is associated with the formation of detached TRAP(+) mononuclear cells and giant osteoclasts in long bones and jawbones (Kuroshima, Go, & Yamashita, 2012; Weinstein, Roberson, & Manolagas, 2009). These nonattached TRAP(+) mononuclear cells induced by the long‐term bisphosphonate therapy are expanded rapidly in response to oral trauma. It is not clear yet if these nonattached mononuclear cells and osteoclasts contribute to the development of ONJ. It is also unclear yet how osteoclasts in the alveolar bone respond differently to denosumab or other antiresorptive agents. Other less potent antiresorptive agents such as estrogen and calcitonin have not been reported to be associated with ONJ (Yamashita & McCauley, 2012). It is likely that the level of resorption inhibition affects the development of ONJ, hence investigating the impact of those antiresorptive agents on osteoclasts in the jawbone in comparison with other bones may provide valuable insights into the ONJ pathogenesis.

### 5.2 Periodontitis

Periodontitis is a chronic inflammatory bone disease characterized by loss of alveolar bone height. Periodontitis is caused by excessive bone resorption due to activation of osteoclasts by pathogenic oral bacteria and subsequent inflammation. Particularly, *Porphyromonas gingivalis* (*P*. *gingivalis*), *Tannerella forsythensis* (*T*. *forsythensis*), and *Treponema denticola* (*T*. *denticola*), known as the “red complexes” in a severity‐ranked hierarchy of disease‐causing bacteria, are considered as periodontal pathogens (Socransky & Haffajee, 2005). Administration of *P*. *gingivalis* into the oral cavity of mice leads to alveolar bone loss associated with increased osteoclastic bone resorption (Zhang, Ju, Rigney, & Tribble, 2014). Interestingly, multiple bacterial infections induce greater alveolar bone resorption than single bacterial infections (Kesavalu et al., 2007). RANKL system plays a critical role in bacteria‐induced bone loss. It was found that increased RANKL expression in gingival tissues is positively correlated with the number of *P*. *gingivalis* in patients with periodontitis (Wara‐aswapati, Surarit, Chayasadom, Boch, & Pitiphat, 2007). It has been suggested that B and T cells are the primary sources of RANKL in the bone resorptive lesion of periodontitis (Kawai et al., 2006; Teng et al., 2000). Osteoblasts, PDL cells, gingival epithelial cells and fibroblasts, and cementoblasts also express RANKL and modulate osteoclastogenesis (de Vries et al., 2006; Fujihara et al., 2014; Huynh, Everts, Pavasant, & Ampornaramveth, 2017; Kikuchi et al., 2001; Tang et al., 2011; Yamaji et al., 1995). The bacterial products such as lipopolysaccharide (LPS) promote proinflammatory cytokine productions such as TNF‐α and IL‐1 from immune cells and activate RANKL expression in osteoblasts. The primary cellular receptor that detects and interacts with LPS is toll‐like receptor 4 (TLR4; Chow, Young, Golenbock, Christ, & Gusovsky, 1999). It has been shown that TNF‐α secreted via LPS/TLR4 signaling regulates osteoclastogenesis in RANKL‐primed macrophages (AlQranei, Senbanjo, Aljohani, Hamza, & Chellaiah, 2021). In addition, TLR2 which primarily recognizes peptidoglycans and lipoteichonic acid of gram‐positive bacteria is also involved in the response to *P*. *gingivalis* (Burns, Bachrach, Shapira, & Nussbaum, 2006). It has been shown that TLR4‐deficient mice exhibit higher levels of RANKL in response to *P*. *gingivalis*, whereas TLR2&4‐deficient mice exhibit no change in RANKL levels, suggesting that TLR2 signaling is also involved in the RANKL‐mediated bone loss. Although RANKL is required for LPS‐induced osteoclastogenesis, inhibition of TNF‐α signaling, but not OPG, blocks the enhancing effects of LPS in osteoclastogenesis (Zou & Bar‐Shavit, 2002), suggesting that enhancing activity of LPS in osteoclastogenesis is likely mediated through inflammatory cytokines but not through RANKL signaling.

It has been demonstrated that oral bisphosphonate improves periodontal health and alveolar bone turnover in postmenopausal women (Akram et al., 2017). Also, local administration of bisphosphonates improves periodontal pocket and alveolar bone defect depths (Arena et al., 2022), suggesting the potentially useful effects of bisphosphonates in preventing bone loss in periodontitis. In an experimental periodontitis mouse model, intraperitoneal administration of anti‐RANKL antibodies prevents alveolar bone loss, and the inhibitory effects are greater than bisphosphonates (Kuritani et al., 2018), suggesting that denosumab may be more useful for preventing alveolar bone loss in periodontitis. Cathepsin K (Ctsk) inhibitors also exhibit potential in the treatment of periodontitis. For instance, local administration of adeno‐associated virus (AAV) expressing *Ctsk* shRNA effectively inhibit both inflammation and osteoclastic bone resorption in *P*. *gingivalis*‐induced periodontitis (Chen et al., 2016). Therefore, local delivery of CtsK inhibitors may minimize adverse effects associated with this class of drugs and provide new treatment options for bone loss in periodontitis. These results suggest that antiresorptive agents seem to be effective in improving periodontitis; however, it is unclear if systemic and local administration of these drugs to treat periodontitis increases the incidence and severity of ONJ. Since periodontal disease and dentoalveolar surgery are the common risk factors for developing ONJ (Hallmer et al., 2018; Wan, Sheeley, Somerman, & Lee, 2020), these medications should be used with caution in patients with periodontitis.

### 5.3 Cherubism

Cherubism is a rare autosomal dominant disorder caused by mutations in the SH3BP2 gene (Ueki et al., 2001). This disease is characterized by excessive bone resorption and accumulation of fibro‐osseous lesions containing TRAP‐positive multinucleated osteoclast‐like giant cells in the maxilla and the mandible including the alveolar bone (Papadaki et al., 2012). The expansion of the fibro‐osseous lesions leads to facial swelling and often interferes with normal tooth development (Roginsky, Ivanov, Ovtchinnikov, & Khonsari, 2009). It has not been considered that Cherubism is an inflammatory bone disorder; however, animal studies have shown a possibility that cherubism is indeed an inflammatory bone disease (de la Fuente, Kumar, Lu, & Geha, 2006; Ueki et al., 2007). It was found that *SH3BP2* mutations in Cherubism result in the formation of hyperactive osteoclasts via activation of NFATc1 (Lietman, Kalinchinko, Deng, Kohanski, & Levine, 2006). In the Cherubism knock‐in (KI) mouse models (*Sh3bp2* ^KI/KI^) in which proline‐to‐arginine (P416R) missense mutation is introduced in the mouse *Sh3bp2* gene, hyperactive macrophages produce high amounts of TNF‐α that drive systemic inflammation and stimulate secretion of RANKL and M‐CSF from stromal cells, leading to excessive osteoclastic bone resorption (Ueki et al., 2007). Although TNF‐α plays a key role in disease pathogenesis in mice, anti‐TNF‐α therapy resulted in no significant improvement in Cherubism patients (Hero et al., 2013; Pagnini et al., 2011), suggesting that the mechanism underlying human cherubism may be different from that of mice. It has been considered that osteoclasts are the only bone‐resorbing cells. Interestingly, however, it was found that activated macrophages can resorb bone matrix even in the absence of osteoclasts in the Cherubism mouse (Kittaka et al., 2018). This non‐canonical bone resorption in mice provides new insights into the mechanism and treatment of inflammatory bone loss.

### 5.4 Orthodontic tooth movement

**FIGURE 6:**
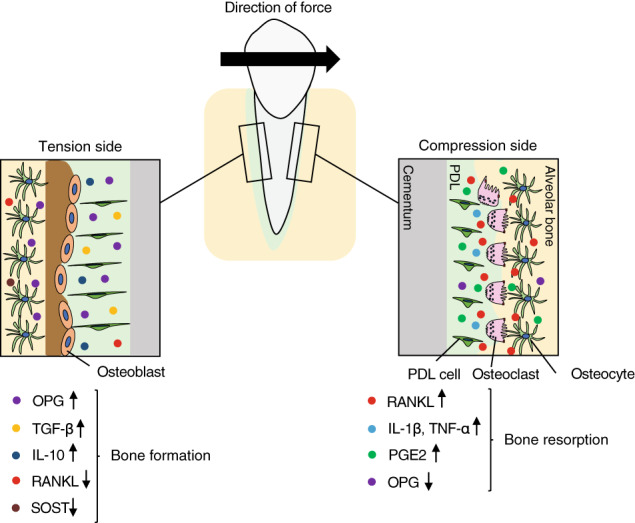
Schematic representation of alveolar bone remodeling during orthodontic tooth movement. The compressed side of PDL is called the compression side and the side where the PDL is pulled is called the tension side. Osteoclasts resorb bone matrix on the compression side to create space for tooth movement, while new bone formation occurs at the tension side. During orthodontic tooth movement, RANKL, IL‐1β, TNF‐α, and prostaglandin (PG)E2 secreted from cells in the PDL or osteocytes on the compression side control osteoclastic bone resorption, while OPG, TGF‐β, IL‐10, and sclerostin (SOST) on the tension side control osteoblastic bone formation

Orthodontic tooth movement is a well‐coordinated biological process that induces alveolar bone remodeling by selectively applying sustained forces to teeth. During orthodontic tooth movement, bone resorption by osteoclasts occurs at the compression side of the tooth socket, compressing PDL fibers, and osteogenesis by osteoblasts occurs at the tension side of alveolar bone (Vavidovitch, 1979; Figure 6). Osteocytes and cells in the PDL play an important role in orthodontic tooth movement‐induced alveolar bone remolding by producing local factors to regulate the cellular activity of osteoclasts and osteoblasts. It was found that ablation of osteocytes reduces osteoclast numbers at the compression side and inhibits orthodontic tooth movement (Matsumoto et al., 2013). Additionally, deletion of *Rankl* in osteocytes leads to reductions of osteoclast cumbers and orthodontic tooth movement (Shoji‐Matsunaga et al., 2017). It was also found that RANKL produced from the PDL cells at the compression side facilitates bone resorption and experimental tooth movement (Kim et al., 2007; Shiotani et al., 2001). Furthermore, HVJ‐envelope‐vector‐mediated local transfer of *Rankl* gene to the periodontal tissue promotes osteoclastic bone resorption and accelerates tooth movement in rats (Kanzaki et al., 2006). By contrast, the transfer of *Opg* gene inhibits RANKL‐mediated osteoclastic bone resorption and prevents tooth movement (Shoji‐Matsunaga et al., 2017), suggesting a critical role of the RANKL/OPG system in orthodontic tooth movement. Also, orthodontic forces increase the expression of IL‐1β and TNF‐α in the PDL (Garlet, Coelho, Silva, & Garlet, 2007), which may amplify RANKL‐mediated osteoclastogenesis. Prostaglandin (PG)E2 released from PDL cells and osteoblasts is also one of the important chemical mediators of compressive force‐induced alveolar bone resorption (Kanzaki, Chiba, Shimizu, & Mitani, 2002; Sanuki et al., 2010). Clinical studies showed that administration of PGE2 into the periodontal tissue accelerates orthodontic tooth movement (Yamasaki et al., 1984). In addition, local administration of Vitamin D3 or PTH to the experimental tooth movement model increases osteoclastogenesis and accelerates tooth movement (Soma et al., 2000; Takano‐Yamamoto, Kawakami, & Yamashiro, 1992). A recent study suggested that the Hippo signaling pathway also plays a vital role in the mechanical signal transduction in PDL cells (Wu, Ou, Liao, Liang, & Wang, 2019). It was found that Hippo signaling component, TEAD1 decreases in response to compressive force stimuli, which contributes to the reduction of OPG and thereby promotes osteoclastogenesis (Wu et al., 2019). These results suggest that the Hippo signaling pathway and inflammatory cytokines are potential targets for orthodontic treatment by increasing osteoclastic bone resorption. By contrast, tensile strain stimulates osteoblast progenitor proliferation and increases the expression of osteogenic factors (e.g., TGF‐β) in the PDL (Van Schepdael, Vander Sloten, & Geris, 2013; Wang, Zhu, & Liang, 2000). It has also been shown that osteocyte production of sclerostin is reduced on the tension side (Nishiyama et al., 2015). In addition to these changes, IL‐10 increases on the tension side, which likely increases OPG and decreases RANKL production to suppress osteoclastic bone resorption (Garlet et al., 2007; Liu, Yao, & Wise, 2006). However, it is unclear whether osteoblastic bone formation on the tension side contributes to a driving force of orthodontic tooth movement.

### 5.5 Osseointegration of dental implants

Osseointegration is defined as a direct structural and functional connection between bone and the surface of a dental implant, used to replace lost dentition (Branemark et al., 1977). The remodeling process around the implant is critically important for stability and survival of the implant (Minkin & Marinho, 1999). After the initial inflammatory phase which follows implant placement, osteoclasts begin peri‐implant bone resorption followed by bone formation to achieve osseointegration (Futami et al., 2000). After the conventional achievement of osseointegration, osteoclasts continue to participate in bone remodeling and newly formed woven bone around the implants is replaced by lamellar bone (Haga et al., 2009). However, excessive osteoclast activity causes failed osseointegration and decreased implant stability (Milinkovic et al., 2021). For instance, aseptic loosening of dental implants has been attributed to inflammatory bone resorption around implants due to titanium (Ti) debris and particles (Eger, Sterer, Liron, Kohavi, & Gabet, 2017). It has been shown that activation of TLRs, especially TLR2 and TLR4, plays a critical role in wear particle‐induced aseptic loosening of orthopedic implants (Bi et al., 2001; Greenfield, Beidelschies, Tatro, Goldberg, & Hise, 2010). These TLRs interact with adaptor protein MyD88, triggering the activation of NF‐κB and production of inflammatory cytokines, leading to osteoclastic bone resorption (Gu, Shi, & Yang, 2012; Pearl et al., 2011). Activation of the NLR family pyrin domain containing 3 (NLRP3) inflammasome also plays an essential role in wear debris‐triggered immune response in macrophages as particle‐induced IL‐1β secretion is dependent upon activation of the NLRP3 inflammasome (Jamsen et al., 2020). Indeed, blocking IL‐1β, IL‐6, or TNF‐α systemically or locally around titanium implants prevents Ti particle‐induced osteolysis (Eger et al., 2018), suggesting that the inflammatory response to implant insertion plays a critical role in the activation of osteoclastic bone resorption.

Mechanical stress on the dental implant provides positive or negative consequences for bone tissue. Under normal functional load, osseointegration is enhanced around the dental implant (Berglundh, Abrahamsson, & Lindhe, 2005; Chavarri‐Prado et al., 2020). While loading increases osteocyte density, osteoclast activation does not change between loaded and unloaded peri‐implant bone (Assenza et al., 2003; Iezzi et al., 2020; Uto et al., 2017), suggesting that osteoclasts might not play a major role in osseointegration of loaded‐implants. Excessive mechanical load on the peri‐implant bone has been thought to be one of the factors that cause bone resorption surrounding the implant; however, it is still controversial if occlusal overload itself leads to implant failures (Chang, Chronopoulos, & Mattheos, 2013; Heitz‐Mayfield et al., 2004; Isidor, 2006). Indeed, implant overloading in an uninflamed peri‐implant environment has a positive effect on the bone‐to‐implant contact while overloading accentuates peri‐implant bone loss in the presence of inflammation (Kozlovsky et al., 2007), suggesting that controlling both peri‐implant inflammation and occlusal load is essential for implant longevity.

As anti‐resorptive agents are effective for treating osteolytic conditions including Paget's disease and osteoporosis, bisphosphonate‐coating to the implant surface improves the early fixation of implants (Abtahi, Tengvall, & Aspenberg, 2012; Tengvall, Skoglund, Askendal, & Aspenberg, 2004; Yoshinari, Oda, Inoue, Matsuzaka, & Shimono, 2002). It was also found that covalently immobilized OPG‐Fc fusion protein on titanium surfaces improves osseointegration by preventing osteoclast formation (Makihira et al., 2010). However, these anti‐resorptive agents must be used with caution as dentoalveolar surgery is considered as a high‐risk factor for ONJ (Barasch et al., 2011). Currently, many studies focus on evaluating the effects of proteins and peptides used to coat surfaces on the biological properties of the implant. Bone morphogenetic proteins (BMPs) are clinically approved osteogenic agents to induce bone regeneration by the US FDA. However, the high doses of BMPs needed can produce unwanted side effects, leading to difficulties in clinical application (James et al., 2016; McClellan, Mulconrey, Forbes, & Fullmer, 2006; Woo, 2012). One of the adverse effects is enhanced osteoclastogenesis as BMPs have direct impact on osteoclast differentiation (Huntley, Jensen, Gopalakrishnan, & Mansky, 2019; Jensen et al., 2010; Omi, Kaartinen, & Mishina, 2019). It has been shown that coating the surface of implants with low dose of BMPs can enhance bone formation and improve osseointegration (Hunziker, Enggist, Kuffer, Buser, & Liu, 2012; Liu, Enggist, Kuffer, Buser, & Hunziker, 2007; Liu, Huse, de Groot, Buser, & Hunziker, 2007). Further clinical studies evaluating the efficacy of BMP‐coated implant surface on implant osseointegration are needed. In addition to implant surface chemistry, the topographical structure of materials affects osteoclast differentiation and function (Davison et al., 2014; Makihira, Mine, Kosaka, & Nikawa, 2007; Webster, Ergun, Doremus, Siegel, & Bizios, 2001). For instance, microstructured Ti surfaces enhance osseointegration by increasing OPG production in vitro (Schwartz, Olivares‐Navarrete, Wieland, Cochran, & Boyan, 2009). On the other hand, enhanced wettability of nanophase ceramics by imparting surface grain size with less than 100 nm stimulates osteoclast activation and resorption (Webster et al., 2001). Additionally, titanium surfaces with increasing surface roughness increase TRAP expression in osteoclasts through the enhanced expressions of RANK and TRAF6 (Makihira et al., 2007). It is likely that successful osteoclast differentiation on the rough implant surface attracts osteoblasts via a coupling mechanism, which, in turn, induces bone formation around the implant (Brinkmann, Hefti, Schlottig, Spencer, & Hall, 2012). In addition to the microscopic design, macroscopic designs including body design and thread geometry are important factors affecting osseointegration of dental implants (Steigenga, al‐Shammari, Nociti, Misch, & Wang, 2003).

To achieve successful use of dental implants in clinical setting, the balanced activities of osteoclasts and osteoblasts are essential. Therefore, understanding the mechanism of osteoclast interactions with dental implants provides new insights to design suitable implant surfaces and materials for alveolar bone regeneration. Furthermore, recent technological advances in tissue engineering approaches offer a new type of dental implants called “bio‐hybrid implants” that provide PDL and cementum attachment on the surface of implants and restore the physiological function of the tooth and periodontium (Lee et al., 2017; Nakajima et al., 2016; Oshima et al., 2014). It would be interesting to know how osteoclasts respond to the bio‐hybrid implants and contribute to alveolar bone remodeling. This technology will lead to significant advancement in the development of a next‐generation therapeutic in tooth and periodontal tissue regeneration, which can preserve the joy of great meals.

## 6 CONCLUSION

Bone resorption by osteoclasts is an essential process to initiate bone remodeling and maintain bone homeostasis. Recent technological advances in live imaging and lineage‐tracing studies in bone have provided new insights into osteoclast biology and opportunities for therapeutics targeting osteoclast functions to treat bone diseases. The alveolar bone exhibits anatomically and functionally unique characteristics compared to axial and appendicular skeletons. Therefore, it is crucial to evaluate the function of osteoclasts in alveolar bone remodeling with advances in technology, which ultimately contributes to understanding the mechanisms underlying jawbone‐specific diseases and overcoming the limitations of current therapies and bone regeneration applications. More comprehensive analyses of the cellular and molecular mechanisms of alveolar bone remodeling will certainly provide more options in clinical dentistry.

## CONFLICT OF INTEREST

All authors declare no conflicts of interest.

## Data Availability

The data that support the findings of this study are available on request from the corresponding author. The data are not publicly available due to privacy or ethical restrictions.
